# A Section of the Future Imperial War Museum

**Published:** 1918-05-18

**Authors:** 


### WOMEN'S WORK IN THE WAR.

### A Section of the Future Imperial War Museum.

FEW people, probably, know of the steady work that is being carried on to preserve a record of the present war. In the early days, when no one imagined the magnitude to which the struggle would reach, many detailed records were not preserved, and many objects that would now have interest and value were thrown away. All the more essential is it that while Armageddon continues the patient behind-the-lines work necessary to provide materials for history shall go on, and treasures for the future Imperial War Museum shall be stored up. In most departments this is steadily being done. Major Brereton, R.A.M.C., is working on at the medical history of the war, munition factories are preserving specimens of successive inventions, models are made of different types of artillery and weapons. A year's work upon the part played by women throughout has already resulted in a great accumulation of records and specimens.

This work is at present in the hands of a Women's Work Sub-Committee, consisting of the Hon. Lady Norman, C.B.E., Chairman; Hon. Lady Haig, Lady Mond, Lady Askwith, C.B.E., Mrs. Carey Evans (*née* Lloyd George), Miss Durham, C B.E., Chief Woman Inspector, Ministry of Labour Employment Department; Miss Monkhouse, M.B.E., Ministry of Munitions; Miss Conway, Hon. Secretary.

Where the future museum will be housed is a point not settled, but obviously a large space will be required. Meantime the work of the Women's Section is carried on at 9 Queen Anne's Gate, and anyone having treasures of national value to offer or a communication of importance to make should write to that address.

Records of events will be kept in some accessible library form. Statuettes of some specially distinguished women will be made; some of these are, in fact, already in existence. Others will be represented by portraits, and, of course, framed photographs representing many of the doings of women will be in evidence. It is proposed to preserve sets of the clothing used for different occupations. Future generations will find interest in the uniforms of the Wrens and the W.A.A.C.s, and even in the trousers and blouses of the girl window-cleaners of to-day.

As might be expected, hospital work comes into special prominence, and a *questionnaire* framed by Lady Mond, whose experience in her own hospital was especially helpful in the selection of points for inquiry, was sent out widely in England and France. When it is realised that information is wanted on women's labours in some 2,000 hospitals in the United Kingdom alone, the mass of information to be collected becomes dizzying. Such work as that of the Scottish Women's Hospitals or the English units in Serbia will especially be represented; one of the portrait statuettes already made is a likeness of Dr. Elsie Inglis. Models of hospital planning and appliances will hereafter possess much interest. Women carpenters and other workers abroad, including those in canteens and rest-huts, will have much to record, and the romance of such adventures as those of the Cellar House at Pervyse will not fade. Work for war refugees already includes such touching trophies as letters of gratitude from Belgian children, lace and embroidery presented by Belgians. Women's work in munition factories, agriculture, and engineering will furnish many specimens. There will also be some beautiful exhibits of embroidery in which women have instructed wounded soldiers; the Royal arms have already been exquisitely worked on satin for this purpose. Decidedly the Women's Section will not be wanting in interest.

